# Integration of conductive reduced graphene oxide into microstructured optical fibres for optoelectronics applications

**DOI:** 10.1038/srep21682

**Published:** 2016-02-22

**Authors:** Yinlan Ruan, Liyun Ding, Jingjing Duan, Heike Ebendorff-Heidepriem, Tanya M. Monro

**Affiliations:** 1 ARC Centre of Excellence for Nanoscale BioPhotonics, Adelaide, SA 5005, Australia; 2 Institute for Photonics and Advanced Sensing, The University of Adelaide, Adelaide, SA 5005, Australia; 3 National Engineering Laboratory for Fibre Optic Sensing Technology, Wuhan University of Technology, Wuhan, 430070, China; 4 School of Chemical Engineering, The University of Adelaide, Adelaide, SA 5005, Australia; 5 University of South Australia, Adelaide, SA 5001, Australia

## Abstract

Integration of conductive materials into optical fibres can largely expand functions of fibre devices including surface plasmon resonator/metamaterial, modulators/detectors, or biosensors. Some early attempts have been made to incorporate metals such as tin into fibres during the fibre drawing process. Due to the restricted range of materials that have compatible melting temperatures with that of silica glass, the methods to incorporate metals along the length of the fibres are very challenging. Moreover, metals are nontransparent with strong light absorption, which causes high fibre loss. This article demonstrates a novel but simple method for creating transparent conductive reduced graphene oxide film onto microstructured silica fibres for potential optoelectronic applications. The strongly confined evanescent field of the suspended core fibres with only 2 μW average power was creatively used to transform graphene oxide into reduced graphene oxide with negligible additional loss. Existence of reduced graphene oxide was confirmed by their characteristic Raman signals, shifting of their fluorescence peaks as well as largely decreased resistance of the bulk GO film after laser beam exposure.

Graphene is comprised of one-atom thick planar sheet of sp^2^-bonded carbon atoms ordered in a two-dimensional honeycomb lattice and has been demonstrated to have ultrahigh carrier mobility, thermal conductivity, and mechanical strength^1^. Graphene also shows remarkable optical properties, including high transparency, high nonlinearity, fluorescence and fluorescence quenching^2^,^3^. Optical fibres have the capacity to excite light and collect signals from a distance and thus are an ideal platform for integration with graphene to exploit applications of its unique optical and electronic properties for a range of practical applications. Some early attempts have been made to manually wrap a small piece of graphene sheet (≈ tens of microns long) onto optical fibre tapers or D-shaped fibres to create all-optical modulator^4^ and four-wave mixing^5^. Due to high conductivity and having a thin layer sheet structure, graphene is also a promising material for integration with fibres along their length to enable a variety of hybrid devices^6^ that require integration of conductors with dielectric waveguides, including those based on surface plasmon resonance^7^, optical modulators^4^,^8^ or even detectors^9^ as well as the detection of chemicals including glucose and gases^10^. However, it is challenging to directly integrate graphene into long length of the fibres due to difficulty in manipulating graphene in form of flakes. Even if this is successfully achieved, the presence of discrete graphene elements along the fibre’s length would lead to significant losses in transmission.

Graphene oxide (GO), a derivative of the graphene, has been used as an alternative to graphene flakes for integration with fibres for ultrafast laser and sensing applications^11^,^12^. GO is graphene sheet modified with oxygen functional groups in the form of epoxy and hydroxyl groups, which makes GO insulating but well dispersive in water^13^. GO has been recognized as a promising precursor for bulk production of graphene-like materials and has similar optical properties to graphene^2^. Thermal and chemical reduction methods have been used to reduce the number of the oxygen-containing groups in the planar surfaces and edges of GO sheets to obtain reduced GO (rGO), which is conductive^14^,^15^.

D-shaped fibres, fibre tapers and fibre facets have been reported as platforms that can support GO films, which are used as saturable absorber enabling ultrafast laser development or as a platform for chemical sensing^11^,^16^,^17^. The GO films were coated onto the fibre endfaces using direct liquid spray or optical coating method^11^,^18^. Due to the limited light-matter interaction length achievable for these fibre platforms^19^, the practical absorption efficiency, nonlinear response or detection sensitivity of these GO films has been limited. In addition, the GO films coated on fibre facets were easily burnt by high power lasers when the GO films were located inside the laser cavities^20^.

We show here that microstructured silica optical fibres (MOFs) with suspended solid cores show advantages over the above-mentioned fibre structures for GO integration. They offer access to extreme fibre properties including tight mode confinement and large power fraction in the evanescent field^21^. Incorporation of graphene or GO onto the cores of MOFs leads to long interaction length of graphene and GO with light, enabling the unique optical and electronic properties of the graphene to be capitalized for optoelectronics applications. In MOFs, the interaction length of light with graphene is only limited by the material absorption of the graphene-like materials themselves and fibre loss.

Here we demonstrate a novel, robust and reproducible method for creating conductive rGO films onto the whole length of the fibre core of suspended core fibres by using their strong evanescent field. We demonstrate that GO sheets can be applied to uniformly cover the core region of the fibres using optical trapping provided by the evanescent field of the fibre mode. For the first time, photoreduction of GO into rGO was achieved in a fibre platform through exposure of the GO films to the evanescent field of the fibres pumped by a picosecond (ps) 532 nm laser. The occurrence of photoreduction was demonstrated from Raman spectroscopy of the GO films and blue shift of the GO fluorescence peak. The creation of rGO films along the whole fibre length demonstrates that optical trapping by the evanescent field is a reliable and easily performed method for integrating conductive materials into a fibre platform, which opens up a new approach for developing optoelectronic devices in optical fibres.

## Results

### GO coating to suspended core fibres

**Figure 1:**
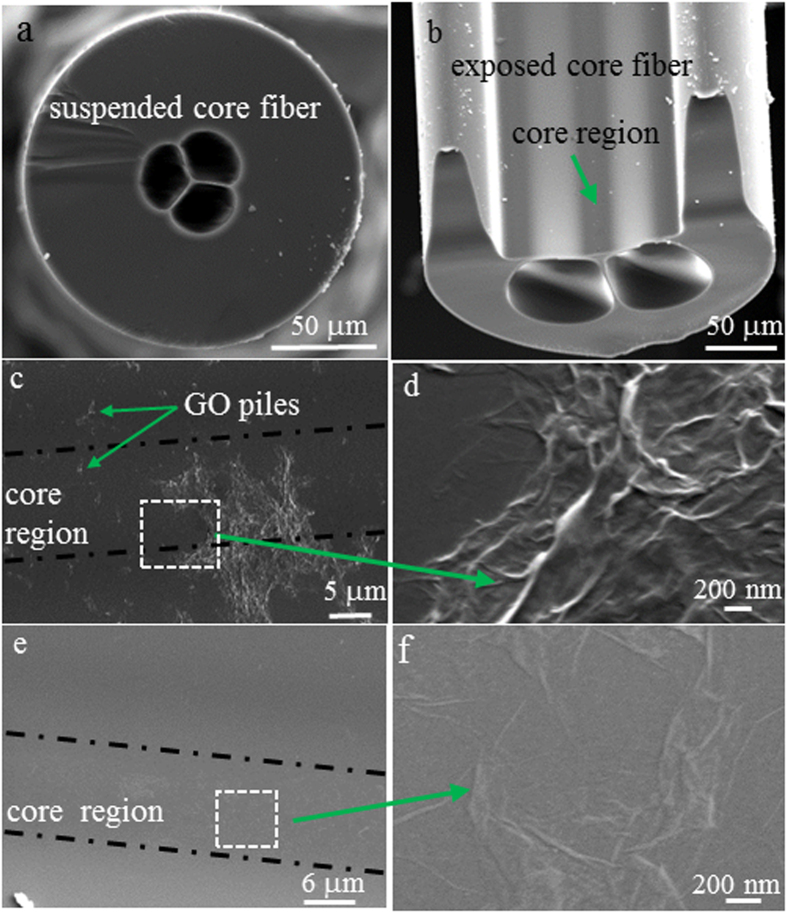
(**a,b**) Are SEM images of the silica SCF and ECF (9 μm core diameter), respectively. (**c**) Is the GO film coated on the exposed core surface of the ECF by filling and baking, (**d**) Is a magnified area in (**c**), (**e**) Is the GO film coated on the exposed core surface of the ECF using 532 nm ps laser and (**f**) is a magnified area in (**e**).

Three silica MOFs were used in the experiments: a suspended core fibre (SCF) with 1.5 μm core diameter, two exposed core fibres (ECFs) with 9 μm and 7.5 μm core diameter, respectively. The SEM images of the SCF and the ECF with 9 μm core diameter are shown in Fig. 1a,b, respectively. The smaller core of the SCFs provides a power fraction in the evanescent field beyond the solid glass core of ~0.4% at 532 nm. The larger core of the ECF (9 μm core diameter) provides about 160 times lower power fraction compared to the SCF but enable easy access to the core for physical and optical characterization of the GO films.

For previous GO coating on fibre endfaces performed by Song’s group^11^, 1550 nm CW laser with guided power level in the range of 100 mW was used for coating^11^. Here no GO coating was observed on the core surface even when 1550 nm CW laser with 1 W power was guided inside them. Thus a ps pulsed 532 nm laser (70 mW maximum average power, 800 ps pulse duration, 10 MHz repetition rate) with high peak power up to 9 W was selected for the GO coating here.

The GO sheets were synthesized via a modified Hummers method^13^ and were homogeneously dispersed in water due to strong electrostatic repulsion between highly negatively charged GO sheets^13^. The average size of the GO sheets in the solution was ~1 μm × 1 μm, and the thickness of the single GO sheet was ~1 nm^22^. A solution with a 0.5 mg/ml concentration was used for our investigation here. The pulsed laser was coupled into the fibre core from one end (hereafter referred to as the proximal end) and the GO suspension was filled into the fibre holes from the other end (hereafter referred to as distal end) by capillary force. We used fibre pieces of ~30 cm length. The length of the fibre section filled with GO dispersion was controlled to be no longer than 20 cm from the distal end to avoid GO dispersion flowing to the proximal end of the fibre.

After filling, the output power of the fibre at the distal end was monitored while the input power at the proximal end was slowly increased. For the SCF with 1.5 μm core diameter, the output power started to decrease with time when the input power was increased to about 500 μW, corresponding to 2 μW average optical power in the evanescent field. This phenomenon indicated an increase in fibre loss, which in turn demonstrated that the GO sheets were coated onto the fibre core, resulting in additional fibre loss. After certain controlled time (25–60 mins), the laser was switched off and the remaining GO suspension was pumped out of the fibre holes, followed by pumping milli-Q water through the holes to clean them. This ensured only the GO sheets coated by the optical trapping remained on the core surface. For comparison, we also used a thermal approach to create a GO film onto the core of a SCF piece. The fibre was first filled with the GO dispersion through capillary force, and then baked at 60 °C for one hour to allow the water to evaporate, and the GO films to form on the internal surfaces of the SCFs. In addition, a third SCF sample was prepared by filling the GO dispersion into the SCF for 20 cm long and the GO stayed inside the holes of the SCF for one hour without coupling any light into this fibre. After one hour, this sample was cleaned and dried using the same method as described above.

For the ECF with 9 μm core diameter, higher average output power of 12 mW achieved by using the maximum laser power was used to coat GO film onto the fibre core surface. This corresponded to 0.3 μW average optical power in the evanescent field, ~7 times lower than that used for GO coating of the SCF discussed above. Thus a longer laser exposure time of 80 mins was chosen for GO coating of the ECF. Since we also used capillary force to fill the GO suspension into the ECF, both the two holes and the open channel of the ECF were filled with GO by capillary force for coating. For comparison, we also used the same filling and baking procedure described above to coat the core of the ECF with GO. As shown in Fig. 1c,d, the GO film formed via baking spread over the whole open channel of the ECF. The film contained a significant number of GO piles due to folding of the GO sheets. By contrast, the GO film formed by using laser irradiation was invisible from the core surface (Fig. 1e,f) probably due to the film consisting of a small number of GO layers that were attached on the core relatively flatly. In some other positions, folded GO sheets were visible (Fig. 1e,f), and were observed to be only located on the core region, indicating that they were coated by the evanescent field of the guided mode of the fibre.

To date three possible mechanisms for optical coating of GO films onto fibres have been reported: optical trapping, thermally induced convection flow and thermophoresis^11^. For the small core SCF, 500 μW output power corresponds to approximately 2 μW average optical power and 0.25 mW peak power in the evanescent field, which extends ~40 nm away from the core surface determined by our modelling. This power has the same order of magnitude as the minimum power of 1 mW required to achieve stable trapping of graphene using conventional free space beam^23^. During the evanescent field induced GO coating, the laser induced heating for the water surrounding the surface of the fibre core region can be estimated from ref. 24, where the local water heating for near infrared (1064 nm) was measured to be ~7.9 K/W. The average optical power of 2 μW in the evanescent field used here for the GO coating of the SCF would lead to a temperature increase <<1.6 × 10^−5 ^K since the water absorption at 532 nm is 5 orders of magnitude lower than that at 1064 nm^25^,^26^. Thus laser induced heating of the water surrounding the core surface for both types of the fibres is negligible^23^, and thermal convection and thermodiffusion effects are expected to be relatively weak in our fibres compared to the coating of GO films on fibre facets using a CW source with tens of mWs power^11^. Thus optical trapping is likely to be the main driving force for coating GO films onto the core surface of the SCFs and ECFs studied here. Theoretical and experimental research on optical trapping of graphene by laser beams^23^ have found that the GO sheets with large optical and shape anisotropy experienced larger rotation motion during optical trapping. In addition, the optical torque force acting on the GO sheets was found to align the GO sheets orthogonal to the light polarization direction^23^. Since the guided modes of the fibres used here are essentially polarized in transverse direction, the GO sheets with small numbers of the layers were coated flatly on the fibre core.

In order to determine the reason for the different effect of the 532 nm ps laser (enabling GO coating) and the 1550 nm CW laser (no GO coating achieved), we analyzed the optical forces exerted on the GO sheets. The scattering force is proportional to the light intensity of the evanescent field of the fibres and propels the particles along the fibre, while the gradient force is proportional to the spatial intensity gradient and traps the GO sheets against the fibre core^27^. 1 W output power of the 1550 nm CW laser from the SCF endface corresponds to 3.4 × 10^6 ^W/cm^2^ intensity of the evanescent field, which is 3000 times higher than the peak intensity of the evanescent field for 500 μW output power of 532 nm ps laser from the same fibre. This means the 1550 nm laser exerted much larger scattering force on the GO along the fibre axis. Meanwhile our modelling showed that the spatial intensity gradient of the 532 nm laser with 500 μW output power is 4 times larger than that of the 1550 nm laser with 1 W output power. Thus, we conclude that the significantly smaller propelling force and the larger gradient force exerted by the 532 nm ps laser enabled the GO to be coated onto the core.

### Raman charaterisation and identification of the crystallite size of the GO film

**Figure 2:**
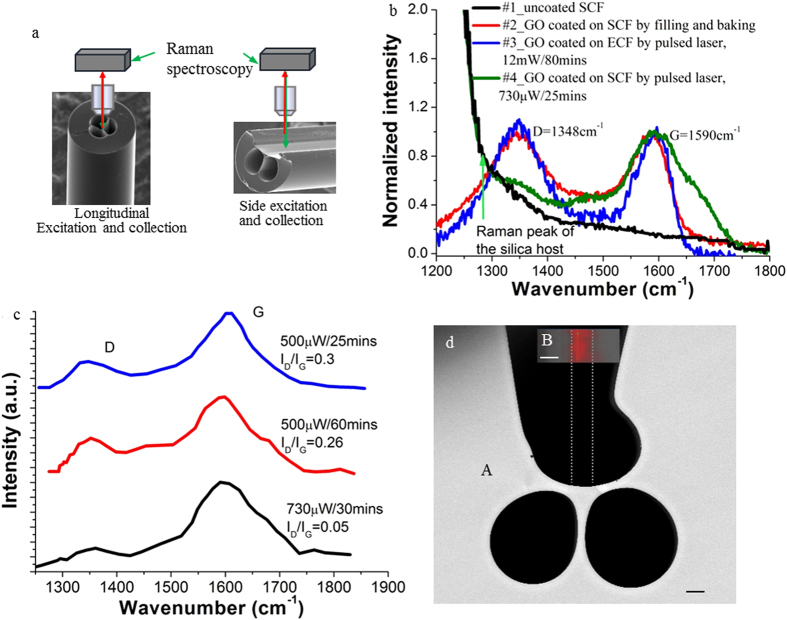
Raman characterization of the GO film coated on the fibre core surface. (**a**) Sketched setup to measure Raman signals of the MOFs. Longitudinal excitation and collection for the SCFs, side excitation and collection for the ECFs. (**b**) Normalized Raman signals detected from the fibres with and without GO coating. Initial fibre output power and time for GO coating are indicated for #3 and #4 fibres. (**c**) Raman peaks of the GO films coated on the SCFs using different laser coating conditions. (**d**) Raman mapping of the region around the exposed core surface of the ECF with 7.5 μm core diameter after coating using laser irradiation. A is the SEM image of the fibre cross-section, B shows the intensity image, taken from the top, of the G Raman peak for the region around the core surface. The high-intensity red colour area indicates the presence of a GO film. The width of this area coincides with the core diameter shown by the vertical dashed lines. The scale bar is 5 μm.

Raman microscopy was used to characterize the GO films coated on the SCF and ECF. We excited and detected the Raman signal by using a 532 nm laser diode from two different directions: longitudinal direction for the SCF and side direction for the ECF as shown in Fig. 2a. Figure 2b shows the Raman spectra of the four fibre pieces investigated: uncoated SCF (#1), SCF coated with GO using filling and baking (#2), ECF and SCF coated with GO using pulsed laser irradiation (#3 and #4). All the GO coated fibres show two characteristic Raman peaks at around 1348 cm^−1^ and 1590 cm^−1^, corresponding to the well-defined D and G bands of GO. The G band is related to the vibration mode of sp^2^ carbon, indicating the degree of the graphitization, whereas the D band is associated with partially disordered structures of sp^2^ clusters^28^,^29^. For the GO coated ECF with 9 μm core diameter, longitudinal excitation and collection to measure the Raman spectrum did not lead to any GO Raman signal, which we attributed to the weaker evanescent field of the larger core ECF compared to the smaller core SCF, causing low excitation and collection efficiency of the GO Raman signal. Note that the SCF sample filled with GO solution but without laser guided in the core did not show GO Raman signals, indicating that the evanescent field of the guided mode is necessary for GO to be coated on the fibre core.

For the SCF core coated with GO using filling and baking method (#2 fibre), the D and G Raman peaks showed similar intensity (red curve in Fig. 2b). The same phenomenon was also observed for the 9 μm ECF core coated with GO using pulsed laser (#3 fibre) when the Raman spectrum was measured from the side direction (blue curve in Fig. 2b). For the SCF core coated with GO using pulsed laser with 500 μW output power (#4 fibre), the Raman signal of silica, the fibre host material, became prominent (the green curve in Fig. 2b), while the D and G peaks were still visible but with the intensity of the D band much weaker than that of the G band. The lower intensity of the D band indicates that the quantity of structural defects in the GO film decreased, demonstrating the generation of reduced graphene oxide (rGO). Since the laser induced coating process lasted for more than 25 mins for all the SCFs, we believe that photoreduction including photochemical and/or photothermal reduction occurred since the GO layers were coated onto the core of the SCFs. It is well known that rGO can be formed by laser irradiation of as-prepared GO solutions/films due to partial removal of the oxygen groups (such as hydroxyl C-OH, epoxyl C-O-C and carbonoxyl –COOH) by photoreduction^15^,^29^,^30^. The intensity ratio of *I*_*D*_*/I*_*G*_ for the D and G bands is widely used for characterizing defect quantity in graphitic materials^31^. In order to determine the impact of the laser coating conditions on the ratio of *I*_*D*_*/I*_*G*_, we prepared three samples of GO coated SCFs under different conditions. To separate the Raman signals caused by the GO coating from that of silica, we subtracted the Raman spectrum of the uncoated SCF (#1 fibre) from the Raman spectra of the GO coated SCFs. These different spectra are shown in Fig. 2c. The coating conditions for these three fibres were: 500 μW output power for 25 mins (blue curve), 500 μW output power for 60 mins (red curve), and 750 μW output power for 30 mins (black curve). For the low power of 500 μW, increasing the coating time from 25 mins to 60 mins decreased the ratio *I*_*D*_*/I*_*G*_ from 0.30 to 0.26. Compared to the low power of 500 μW, the higher power of 730 μW reduced the *I*_*D*_*/I*_*G*_ ratio by a factor of 6 to a value of 0.05. This result indicates that higher power is more effective than increased laser irradiation time for removing oxygen groups from the GO film. Note that for the GO film coated onto the ECF core using laser irradiation, even use of the full power of the laser did not result in a reduction of *I*_*D*_*/I*_*G*_, which indicates lack of photoreduction due to the weaker evanescent field of the ECF compared to the SCF (#3 fibre in Fig. 2b).

The in-plane crystallite size of the sp^2^ cluster of rGO determines the electrical conductivity of rGO. More specifically, the conductivity increases with increasing crystallite size due to reduced hopping of carriers between the sp^2^ clusters^31^. The average crystallite size *L*_*a*_ can be calculated by 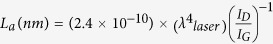, where *λ*_*laser*_ is the excitation wavelength used in the Raman measurement^31^. This equation indicates that the crystallite size and thus conductivity increases with decreased *I*_*D*_*/I*_*G*_ ratio. Chemical reduction of rGO by hydrazine was found to increase *I*_*D*_*/I*_*G*_ ratio, indicating a decrease in the average crystallite size^32^,^33^. By contrast, the use of thiophene to form rGO resulted in a decrease of *I*_*D*_*/I*_*G*_ that indicated the formation of larger crystallites of rGO, which led to enhanced conductivity of the rGO. To the best of our knowledge, the low ratio of *I*_*D*_*/I*_*G*_ ~ 0.05 achieved in our fibre platform is the lowest value achieved so far by photoreduction. Using the above equation, the average crystallite size of the sp^2^ cluster of the GO film of #4 fibre is 380 nm, which is about 20 times larger than that of the GO film coated onto the SCF by filling and baking. This sp^2^ cluster size is about 10 times larger than those reported so far^29^, which promises the rGO film in the SCF to exhibit high conductivity.

We also performed mapping of the Raman signal of GO around the exposed core surface of the ECF with 7.5 μm core diameter after GO coating using 532 nm ps laser with 5.2 mW output power from the fibre and 80 min laser exposure. The Raman image shown in Fig. 2d indicates the GO coated area (red area) is located in the centre of the region around the core surface with uniform thickness and has a width of 6.3 μm, which is close to the diameter of the fibre core (7.5 μm). This demonstrates the GO coating is only formed in the area of the evanescent field of the fibre core, indicating that the evanescent field plays a key role for GO coating.

### Photoreduction induced fluorescence change

Photoreduction was further confirmed by observation of changed fluorescence bandwidth of the GO sheets coated on SCFs using the pulsed laser. Low-energy fluorescence in the red to near infrared wavelength region has been reported for dispersion and solid thin films of as-synthesized GO^34^,^35^. The red emission of GO is associated with oxygen-related structures^34^,^35^. We measured the fluorescence of the GO incorporated into the SCFs using the backward detection method described in ref. 36 and low power (700 μW) laser at 407 nm wavelength for excitation. In addition, we calculated insertion loss of the fibres by measuring the input and output power of the excitation laser in fibre transmission configuration. This enabled the additional fibre loss caused by GO coating to be calculated by subtracting the insertion loss of the fibre with the same length and without GO coating. Note that the high power 532 nm ps laser used for coating is hereafter referred to as coating laser.

**Figure 3:**
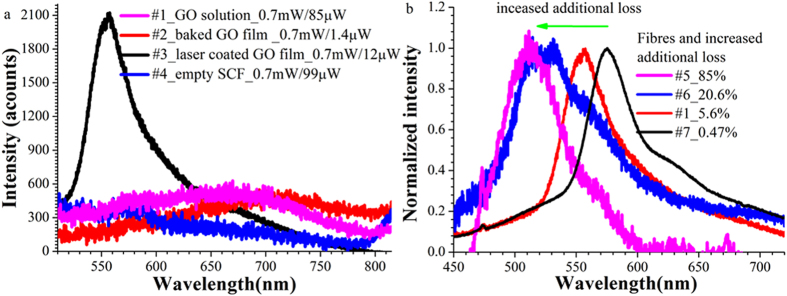
Fluorescence of the GO films coated on the core surface of SCF pieces. (**a**) Fluorescence spectra of the four fibre pieces with or without GO films incorporated. The grating used was 600 g/mm and integration was 1 s. The two power values in the legend are the input and output power of the fluorescence excitation laser at 407 nm wavelength and indicate the fibre loss. For the fibre with the GO coated on the core using laser irradiation, the detected fluorescence became narrower and stronger. (**b**) For the fibres with GO coated using laser irradition, they show blue shifted fluorescence with increased fibre additional loss.

For the as-synthesized GO dispersion filled into the SCF (#1) or GO film coated onto the fibre core surface by filling and baking (#2), we detected weak broadband red fluorescence with a peak position around 680 nm and bandwidth of >170 nm (violet and red curves in Fig. 3a). This fluorescence band had a weak intensity that was only 300 counts higher than that of the background fluorescence of the empty SCF (blue curve in Fig. 3a).

By contrast, the SCF (#3) coated with a GO film using coating laser irradiation displayed a sharp fluorescence spectrum with its peak wavelength blue shifted to 557 nm (black curve in Fig. 3a) and its bandwidth decreased to 46 nm, nearly 4 times narrower than the fluorescence of other fibres (#1 and #2). In addition, this fibre showed 4 times higher peak fluorescence intensity compared to other fibres. As mentioned above, we observed that GO coating induced additional fibre loss. It increased with increasing coating laser power and exposure time. The degree of the blue shift of the fluorescence peaks of the SCFs was found to become larger as the additional fibre loss increased (Fig. 3b). For the #5 fibre with 85% additional loss, its peak fluorescence moved to 512 nm, corresponding to a shift of 63 nm by comparing to that of the 7# fiber with 0.47% additional loss. We hypothesize that with increased coating laser power or time, the GO films, particularly those GO layers firstly coated onto the core surface, were irradiated for an extended time, which may result in a larger proportion of the GO films being reduced to rGO and thus a larger blue shift of the fluorescence peak. Similar fluorescence blue shifts with increased coating laser exposure time of GO dispersions have been reported for different laser systems^34^,^35^,^37^. The blue shift was attributed to photoreduction of the GO to rGO^35^,^37^. XPS measurement by Nemeto group^34^ showed that the photoreduction resulted in an increase of the number of sp^2^ carbon clusters and removal of oxygen-containing functional groups. The increased number of the sp^2^ clusters yielded a narrower emission bandwidth while disordered functional groups caused a broad prominent photoluminence at longer wavelengths^34^. Therefore we assume that similar photoreduction occurred during our coating processes using a 532 nm ps laser. The occurrence of GO fluorescence changes due to photoreduction is consistent with the observed change in Raman signals of the GO films as discussed above.

### Increased conductivity of the GO film by laser exposure

Measurement of the conductivity of a GO film requires an electrical connection to the film surface. However, it is challenging to integrate an electrical connection to the core surface of the SCF. Thus, we investigated electrical transport properties of GO films firstly coated on a coverslip and a conventional single mode fibre (SMF), and then exposed to the same 532 nm ps pulsed laser. We used this method to test our hypothesis that the 532 nm ps laser had the capability to transform GO to conductive rGO.

**Figure 4:**
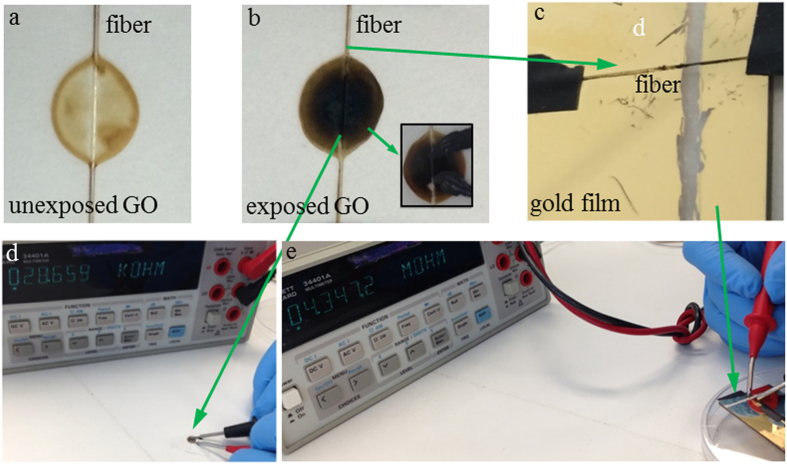
GO film coated on a coverslip. (**a**) Unexposed GO film with a SMF crossing it; (**b**) The GO film was exposed by a 532 nm ps laser with an average intensity of 8 W/cm^2^ for 15 hs, and showed darker color in the center than its edge area. The insert shows the Carbon DAG^®^ used as electrical contact for resistance measurement. (**c**) The fibre in b was positioned to cross two pieces of the gold film for resistance measurement. The GO film on the coverslip showed resistance of 29 kΩ(**d**), and 4.3 MΩ when it was coated on the SMF (**e**).

100 μl of GO dispersion was dropped onto a bare SMF located on a coverslip, which was then dried naturally in a fume hood to allow the GO film to be formed as shown in Fig. 4a. The dry GO film was exposed to the beam of the 532 nm ps laser for a period with an average maximum intensity of 8 W/cm^2^ achieved for a 2 mm beam diameter. As shown in Fig. 4b, it has been found that the exposed area changed its color from yellow to dark gray. This is a sign that rGO was formed^15^. Carbon DAG^®^ was coated at the edges of the exposed black area to create electrical contact for resistance measurement. For as-prepared GO films on coverslips without laser exposure, its resistance was out of the upper measurement limitation of the multimeter (1 × 10^8 ^Ω). After laser exposure, it was found that the resistance of the GO samples decreased with increasing laser exposure time. The GO film with two hours exposure showed 5 MΩ resistance. However, when the exposure time was increased to 15 hs for the sample shown in Fig. 4b, its resistance was decreased to 29 kΩ (Fig. 4d). For the fibre with 125 μm diameter, when it was taken away from the GO film in Fig. 4b, its resistance was measured as 4.3 MΩ by positioning it across two well separated gold pads (1 cm gap) coated on a coverslip (Fig. 4c). The increased resistance of the fibre itself compared to the GO film (Fig. 4d) was due to reduced area of the GO film coated along the fibre surface. The reduced resistance of the GO film on the glass substrate after laser exposure further indicated that photothermal reduction of GO to rGO occurred during exposure of the 532 nm ps laser. For the GO coated SCF fibre with 1.5 μm core diameter, we calculated the average intensity of the evanescent field on the core surface for 500 μW output power to be approximately 980 W/cm^2^, 120 times higher than the maximum intensity of 8 W/cm^2^ achieved for the free space beam with 2 mm diameter. Based on the significantly higher power of the evanescent field of the SCF compared to the free space beam, we conclude that the 532 nm ps laser irradiation via the evanescent field of the SCF created conductive rGO film along the fibre core.

## Discussions

In conclusion, using a pulsed 532 nm picosecond laser with high peak power, we successfully coated GO films onto the core surfaces of small core SCFs and ECFs. Raman spectroscopy showed that the laser irradiation led to a decrease in the D Raman peak intensity of the GO film relative to its G peak due to decreased structural defects, indicating formation of rGO. In addition, after being exposed to the evanescent field of the fibre, the fluorescence band of the GO film on the core surface became narrower and blue shifted. The wavelength shift of the fluorescence peak increased with longer laser exposure time or higher power. These results further confirm the fact that GO has been transformed to rGO due to photoreduction. Exposure of a GO film coated on a coverslip to a 532 nm ps free space laser beam led to significant increase of the film conductivity, which demonstrates that this laser has sufficient intensity to transfer GO to rGO. By comparing the relative intensity of the Raman peaks of the rGO created onto the fibre cores to that of the rGO made by chemical reduction, we found that the former has potential to achieve higher conductivity than the latter.

Our work showed that conductive rGO films were incorporated onto the core surface of the SCF. This signifies a breakthrough for the integration of conductive materials over long lengths of fibres for optoelectronic applications. Further study will be performed to develop a method to directly measure the conductivity of the GO films coated onto the core surfaces, and further optimized processes will be required to improve the conductivity to a practically applicable level.

Although some early attempts have been made to incorporate metals such as tin into fibres during the fibre drawing process^38^,^39^,^40^, these approaches are challenging due to the restricted range of materials that have compatible melting temperatures with that of silica glass. Moreover, metals are nontransparent with strong light absorption, which causes high fibre loss. Here we showed that the guided light has the capability to form rGO along long length of the SCFs without sacrificing the fibre loss when only a small amount of rGO layers are coated. Thus we have shown a new route for incorporating a transparent and conductive material into a fibre platform and add conductivity without compromising the loss of the fibres and thus the potential of a fibre platform for long interaction length between light and rGO can be realized.

For practical applications of the rGO films along the fibres, the increased loss of thick GO films has a detrimental effect on the device performance. Monolayer graphene was shown to be an effective saturable absorber for mode-locking fibre lasers^41^ and used successfully as modulator^4^ and for four-wave mixing^5^. Thus GO films with only one or few layers are sufficient for practical applications, and such thin films cause negligible additional loss as observed in our experiments.

## Additional Information

**How to cite this article**: Ruan, Y. *et al.* Integration of conductive reduced graphene oxide into microstructured optical fibres for optoelectronics applications. *Sci. Rep.* **6**, 21682; doi: 10.1038/srep21682 (2016).
